# Contributions of glucocorticoid receptors in cortical astrocytes to memory recall

**DOI:** 10.1101/lm.053041.120

**Published:** 2021-04

**Authors:** William W. Taylor, Barry R. Imhoff, Zakia Sultana Sathi, Wei Y. Liu, Kristie M. Garza, Brian G. Dias

**Affiliations:** 1 Neuroscience Graduate Program, Emory University, Atlanta, Georgia 30322, USA; 2 Neuroscience Graduate Program, University of Southern California, Los Angeles, California 90007, USA; 3 Developmental Neuroscience and Neurogenetics Program, Division of Research on Children, Youth, and Families, The Saban Research Institute, Children's Hospital Los Angeles, Los Angeles, California 90027, USA; 4 Division of Behavioral Neuroscience and Psychiatric Disorders, Yerkes National Primate Research Center, Atlanta, Georgia 30322, USA; 5 Wallace H. Coulter Department of Biomedical Engineering, Georgia Institute of Technology and Emory University, Atlanta, Georgia 30322, USA; 6 Department of Psychiatry and Behavioral Sciences, Emory University School of Medicine, Atlanta, Georgia 30322, USA; 7 Department of Pediatrics, Keck School of Medicine, University of Southern California, Los Angeles, California 90027, USA

## Abstract

Dysfunctions in memory recall lead to pathological fear; a hallmark of trauma-related disorders, like posttraumatic stress disorder (PTSD). Both, heightened recall of an association between a cue and trauma, as well as impoverished recall that a previously trauma-related cue is no longer a threat, result in a debilitating fear toward the cue. Glucocorticoid-mediated action via the glucocorticoid receptor (GR) influences memory recall. This literature has primarily focused on GRs expressed in neurons or ignored cell-type specific contributions. To ask how GR action in nonneuronal cells influences memory recall, we combined auditory fear conditioning in mice and the knockout of GRs in astrocytes in the prefrontal cortex (PFC), a brain region implicated in memory recall. We found that knocking out GRs in astrocytes of the PFC disrupted memory recall. Specifically, we found that knocking out GRs in astrocytes in the PFC (AstroGRKO) after fear conditioning resulted in higher levels of freezing to the CS+ tone when compared with controls (AstroGRintact). While we did not find any differences in extinction of fear toward the CS+ between these groups, AstroGRKO female but not male mice showed impaired recall of extinction training. These results suggest that GRs in cortical astrocytes contribute to memory recall. These data demonstrate the need to examine GR action in cortical astrocytes to elucidate the basic neurobiology underlying memory recall and potential mechanisms that underlie female-specific biases in the incidence of PTSD.

Recalling important information about salient environmental cues is an integral part of how we navigate our world. Recalling too much, or too little, information about salient environmental cues is a part of the psychopathology of posttraumatic stress disorder (PTSD) (Milad and Quirk 2012). More specifically, the augmented recall of an association between an environmental cue and a traumatic event results in debilitating fear toward the cue, even in the absence of any threat. In contrast, impoverished recall of information that a cue, previously associated with trauma, is no longer a threat also results in debilitating fear toward the cue after it is no longer dangerous. Therefore, one way to mitigate debilitating fear that characterizes PTSD is to understand the neurobiological mechanisms underlying memory recall.

Among many mechanisms, glucocorticoid action via signaling through glucocorticoid receptors (GRs) is an important neurobiological pathway that underlies the recall of salient information. When trauma-associated cues are encountered, the hypothalamic-pituitary-adrenal axis is activated and GR signaling is consequently triggered (McEwen et al. 1988; McEwen 1992; Lupien et al. 2009). Existing literature demonstrates that glucocorticoids and GRs do in fact influence learning, memory, and the recall of learning (Pugh et al. 1997; de Quervain et al. 1998, 2009, 2011, 2017, 2019; Roozendaal 2002, 2003; Conrad et al. 2004; Hui et al. 2004; Donley et al. 2005; Roozendaal and de Quervain 2005; Cai et al. 2006; Soravia et al. 2006; Yang et al. 2006; Roozendaal et al. 2009; Bentz et al. 2010; Blundell et al. 2011; Clay et al. 2011; Nikzad et al. 2011; Roesler 2012; Liao et al. 2013; Wislowska-Stanek et al. 2013; Arp et al. 2016; Reis et al. 2016; Dadkhah et al. 2018; Inoue et al. 2018; Scheimann et al. 2019; Lin et al. 2020). The relationship between glucocorticoids, GRs, learning and memory is complicated and within the literature cited above, one can find examples of GR action being facilitatory as well as inhibitory to learning and memory recall. As expansive as this research is, the influence of GRs on learning, memory, and recall of learning has mostly focused only on GR action in neurons or has ignored cell type specific contributions. While glia are approximately as common as neurons in the nervous system (von Bartheld et al. 2016; von Bartheld 2018), the role of GRs in glial cells on the recall of salient environmental cues has been neglected. More specifically, while astrocytes comprise a significant proportion of the glial cell population (von Bartheld et al. 2016) and express GRs (Vielkind et al. 1990; Bohn et al. 1991), the influence of GRs in astrocytes on memory recall remains largely unappreciated (for one exception, see the Discussion).

Our goal in this study was to determine the influence of GRs in astrocytes on memory recall. To do so, we combined the robust and reliable experimental framework of classical fear conditioning in rodents (Santini et al. 2008; Dias et al. 2014; Bukalo et al. 2015; Keiser et al. 2017; Giustino and Maren 2018; Greiner et al. 2019; Gunduz-Cinar et al. 2019; Venkataraman et al. 2019) with molecular genetic manipulations in the prefrontal cortex (PFC), a brain region critical for the recall of memory (Morgan and LeDoux 1995; Quirk et al. 2000; Mueller et al. 2008; Quirk and Mueller 2008; Giustino and Maren 2015; Rozeske et al. 2015; Maren and Holmes 2016). We first trained mice to associate tone presentations with mild footshocks. After this auditory fear conditioning, we used a CRE-loxP strategy to specifically knock out GRs in astrocytes in the PFC (hereafter termed cortical astrocytes) of these trained mice. We then exposed animals to extinction training: 30 presentations of the tone in the absence of any footshocks. Finally, 1 d after the extinction training, we exposed animals to two presentations of the tone. This experimental timeline allowed us to ask how a lack of GRs in cortical astrocytes influences (1) the recall of the previous aversive association of the tone presentation with the footshock, (2) the extinction of fear that would typically occur during extinction training, and (3) the recall of extinction training allowing us to measure the influence of GRs in cortical astrocytes on the recall of extinction training. Broadly, our results demonstrate that knocking out GRs in cortical astrocytes disrupts fear memory recall in both male and female mice, while only disrupting extinction recall in female mice.

## Results

### Knocking out GRs in cortical astrocytes using a Cre-LoxP strategy

**Figure 1.**
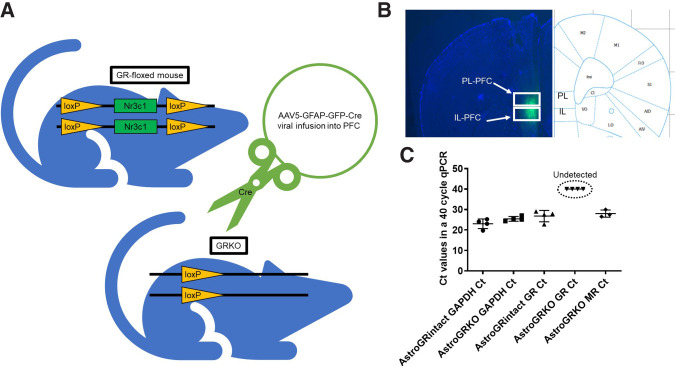
CRE-mediated knockout of GRs in astrocytes in the PFC. (*A*) AAV5-GFAP-GFP-Cre virus was injected into the PFC of GR-floxed mice to induce Cre recombinase-mediated knockout of the *nr3c1* gene in astrocytes in the PFC. (*B*) Representative image of PFC injections spanning both the prelimbic (PL) and infralimbic (IL) subdivisions of the PFC (Allen Mouse Brain Atlas). (*C*) Astrocytes were isolated using FACS and qPCR was performed to determine *nr3c1* gene expression in astrocytes. Transcripts of *gapdh* were detected in both AstroGRKO animals (*C*_t_ = 25.40 ± 0.5903) and AstroGRIntact animals (*C*_t_ = 23.0 ± 1.184). *C*_t_ values for *gapdh* did not differ significantly between the groups (*P* > 0.05). *nr3c1* transcripts were detected only in AstroGRintact animals (*C*_t_ = 26.77 ± 1.386). *nr3c1* transcripts were not detected in AstroGRKO animals, not reaching fluorescent threshold in 40 cycles. *nr3c2* transcripts were detected in AstroGRintact animals (*n* = 3–4/group, ANOVA: *F*_(3,12)_ = 63.44, *P* < 0.0001; post-hoc comparisons: AstroGRIntact GAPDH *C*_t_ vs. AstroGRKO GAPDH *C*_t_, *P* > 0.05, AstroGRIntact GR *C*_t_ vs. AstroGRKO GR *C*_t_ *P* < 0.0001). (*nr3c1*) Gene encoding GR, (*nr3c2*) gene encoding mineralocorticoid receptor.

To knock out GRs in astrocytes in the PFC, we infused either GFAP promoter driven GFP expressing AAVs or GFAP promoter driven Cre + GFP expressing viruses into the PFC of GR-floxed mice (Fig. 1A,B). This strategy resulted either in no perturbation of the GR receptor (AstroGRintact) or Cre recombinase-mediated knockout of the *nr3c1* gene in astrocytes in the PFC (AstroGRKO). We validated the efficacy of our knockout strategy by RT-qPCR after extracting RNA from GFP-expressing astrocytes via FACS sorting. While *nr3c1* transcripts were detected in astrocytes of the AstroGRintact group, no *nr3c1* transcripts were detected in astrocytes of the AstroGRKO mice at the end of the 40 cycles of qPCR (Fig. 1C). Equal amounts of *gapdh* transcripts were detected in the AstroGRintact and AstroGRKO groups (*n* = 4/group, ANOVA: *F*_(3,12)_ = 63.44, *P* < 0.0001; post-hoc comparisons: AstroGRintact GAPDH *C*_t_ vs. AstroGRKO GAPDH *C*_t_, *P* > 0.05, AstroGRintact GR *C*_t_ vs. AstroGRKO GR *C*_t_ *P* < 0.0001) (Fig. 1C). Importantly, we detected *nr3c2* transcripts (encoding mineralocorticoid receptors) in the AstroGRKO group.

### Knocking out GRs in cortical astrocytes disrupts fear memory recall

**Figure 2.**
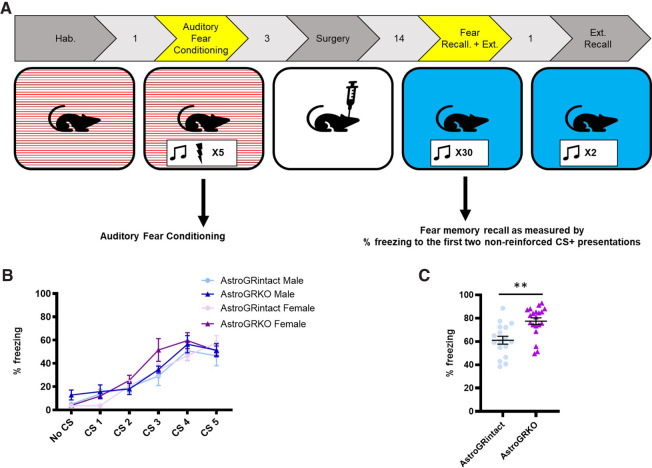
Loss of GRs in cortical astrocytes disrupted normative recall of fear memory. (*A*) Experimental design showing data in this panel depicting auditory fear conditioning and recall of fear memory when GRs were knocked out in cortical astrocytes after animals had been exposed to auditory fear conditioning. (*B*) Prior to GRs being knocked out in cortical astrocytes of AstroGRKO mice (which occurs after fear conditioning), AstroGRKO mice showed no differences in the acquisition of fear during auditory fear conditioning compared with AstroGRintact animals. Data show percent time freezing to the CS+. (*C*) Following auditory fear conditioning and knockout of GRs, AstroGRKO mice froze significantly more to the first two CS+ presentations compared with AstroGRintact mice (two-way ANOVA [sex × GR status]: GR status main effect—*F*_(1,33)_ = 13.30, *P* = 0.0009, sex main effect—*P* > 0.05, sex × GR status interaction—*P* > 0.05). Therefore, data shown are collapsed across sexes. See Supplemental Figure S2 for data split by sex. All data are represented as mean ± SEM.

Prior to GR knockout, all animals underwent auditory fear conditioning in Context A (Fig. 2A). During auditory fear conditioning (CS+ tones paired with footshocks), female and male AstroGRKO mice (female: *n* = 8, male: *n* = 12) show similar acquisition of fear to the CS+ (conditioned stimulus) as AstroGRintact mice (female: *n* = 9, male: *n* = 8) (Fig. 2B). The freezing of both female and male mice did not vary significantly as a function of “GR status,” which was expected as GRs have not yet been knocked out (two way ANOVA [sex × GR status]: *F*_(3,33)_ = 0.719, *P* > 0.05) (please see Supplemental Fig. 1 for data split by sex).

Following auditory fear conditioning, the mice were left to consolidate memory of the conditioning over 3–5 d before receiving intracranial injections of AAV-GFAP-GFP or AAV-GFAP-CRE-GFP into the PFC. To ensure expression of GFP or CRE recombinase, animals were left for 2 wk before being presented with two, nonreinforced CS+ presentations in context B (Fig. 2A). Freezing to these two CS+ presentations is reported as a measure of the recall of fear memory. AstroGRKO mice froze significantly more than AstroGRintact mice (Fig. 2C) (two way ANOVA [sex × GR status]: sex × GR status interaction—*P* > 0.05, sex main effect—*P* > 0.05, GR status main effect—*F*_(1,33)_ = 13.30, *P* = 0.0009; therefore, data collapsed across sexes) (please see Supplemental Fig. 2 for data split by sex).

### Knocking out GRs in cortical astrocytes does not influence extinction of fear, but disrupts recall of extinction training in female but not male mice

**Figure 3.**
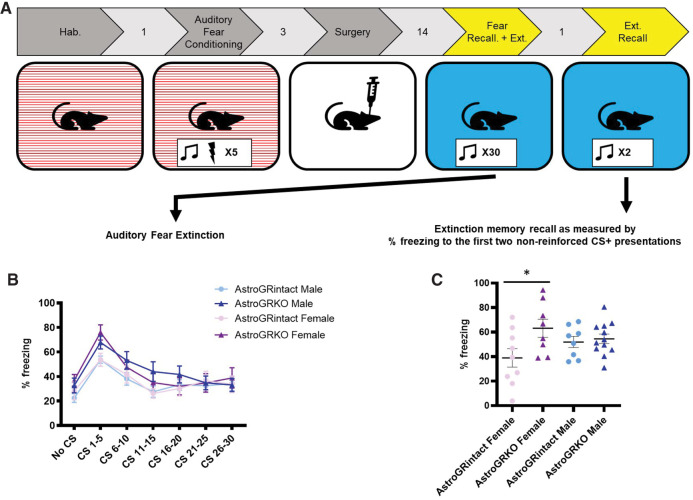
Loss of GRs in cortical astrocytes did not impact fear extinction, but impaired the recall of extinction training in female but not male mice. (*A*) Experimental design showing data in this panel depicting acquisition of extinction during extinction training, and subsequent extinction recall 1 d later, when GRs were knocked out in cortical astrocytes after animals had been exposed to auditory fear conditioning. (*B*) Following GRs being knocked out in cortical astrocytes after auditory fear conditioning and before exposure to extinction training, AstroGRKO mice showed fear extinction to repeated nonreinforced presentations of the CS+ during extinction training that is comparable with AstroGRintact mice. Data show percent time freezing to 30 CS+ presentations (six bins of five CS+ presentations). (*C*) One day after extinction training, AstroGRKO female mice froze significantly more to two CS+ presentations than AstroGRintact female mice, but male AstroGRKO and AstroGRintact mice did not differ in their freezing to the CS+. Data are split by sex because of a statistically significant sex × GR status interaction (two-way ANOVA [sex × GR status]: GR status × sex interaction—*F*_(1,33)_ = 4.149, *P* < 0.05; post-hoc Sidak's multiple comparisons: AstroGRintact vs. AstroGRKO—male *P* > 0.05, AstroGRintact vs. AstroGRKO—female *P* = 0.01). All data are represented as mean ± SEM.

Following auditory fear conditioning and virus expression, the animals were presented with 30 CS+ presentations in Context B during extinction training (Fig. 3A). We found a significant interaction between GR Status and CS+ bins when comparing the freezing of male and female AstroGRKO mice with freezing of AstroGRintact mice during extinction training (two-way ANOVA [GR status × CS]: *F*_(15,165)_ = 2.029, *P* = 0.01). As noted in the previous section, this effect was driven by AstroGRKO mice freezing significantly more to the first few presentations of the CS+ (fear recall) compared with AstroGRintact mice (Fig. 2C). However, notably, AstroGRKO mice showed similar extinction of fear to the CS+ as AstroGRintact mice by the end of the training session (Fig. 3B).

One day after extinction training, the mice were returned to context B and exposed to 2 CS+ presentations without US pairings (Fig. 3A). Freezing to these two CS+ presentations was considered a measure of the recall of extinction training. We found a significant GR Status × Sex interaction, with female AstroGRKO mice freezing significantly more than female AstroGRintact mice, but no differences between male AstroGRKO and male AstroGRintact mice (two way ANOVA [sex × GR Status]: GR status × sex interaction—*F*_(1,33)_ = 4.149, *P* < 0.05; post-hoc Sidak's multiple comparisons: AstroGRintact vs. AstroGRKO—male *P* > 0.05, AstroGRintact vs. AstroGRKO—female *P* = 0.01) (Fig. 3C; please see Supplemental Fig. 3 for data split by sex) (Fig. 3C).

## Discussion

In this study, we combined the robust and reliable experimental framework provided by auditory fear conditioning with molecular genetic-based knockout of GRs in cortical astrocytes to ask whether and how GRs in astrocytes in the PFC impact learning and memory. To do so, we knocked out GRs in cortical astrocytes of female and male mice after CS+ tone + shock fear conditioning. Confirmation of this outcome with FACS and qPCR showed that GRs were successfully knocked out in all infected astrocytes. While this measurement does not provide an estimate for the proportion of astrocytes that remained uninfected by our virus and that would therefore continue to express GRs, our behavior data demonstrate that this number was not sufficient to rescue our observed deficits in memory recall. We found that knocking out GRs in cortical astrocytes of mice after fear conditioning resulted in high levels of fear toward subsequently encountered CS+ tones. We also found, specifically in female mice, high levels of fear toward CS+ tones encountered after subsequent extinction training, during which CS+ tones had been presented in the absence of any footshock. We did not observe these effects in male mice. These results provide strong evidence that GRs on cortical astrocytes play an important role in normative recall of salient environmental events and that there may be sex differences in the role that these GRs play in learning and memory.

Heightened expression of fear toward stimuli previously associated with trauma is a debilitating and highly prevalent dimension of PTSD (Holmes and Singewald 2013; Dias et al. 2015; Ressler 2020). Such heightened fear could be the result of information that links stimulus and traumatic outcome becoming overly consolidated at the time of this juxtaposition. Alternatively, it could be the result of memory about the association being recalled differently when the stimulus is encountered in the future. In our experimental design, we knocked out GRs in cortical astrocytes several days after the auditory fear conditioning and outside the period during which consolidation of the fear conditioning would occur. This timeline leads us to interpret our finding of AstroGRKO mice showing increased fear toward the initial presentations of the CS+ tones much after CS+ tone + shock fear conditioning as evidence for GRs in astrocytes in the PFC being important in the normative recall of fear memories. To dissociate the influences of GRs in cortical astrocytes on consolidation versus recall, future experiments would need to use as yet unavailable conditional approaches to knock down these GRs only at the time of consolidation and for GR expression to recover and be at baseline at the time of recall.

Another highly prevalent dimension of PTSD is the expression of fear toward stimuli that may have been previously associated with trauma but are no longer threatening (Kearns et al. 2012; Milad and Quirk 2012; Maren 2014; Greco and Liberzon 2016). This dimension manifests as heightened fear to the CS+ tones even after extinction training, during which the CS+ tones are no longer paired with a footshock. Such debilitating fear could result from an inability to learn that stimuli are no longer threatening, an inability to consolidate this information, or an inability to recall this information. We found that AstroGRKO female mice show within-session extinction that is indistinguishable from controls. These data argue against an influence of GRs in astrocytes in the PFC on learning that stimuli are no longer threatening within an extinction training session. Notably, 1 d after extinction training, AstroGRKO female mice show increased fear toward CS+ presentations. These findings come with the important caveat that they do not allow us to distinguish whether knocking out GRs in cortical astrocytes impairs the consolidation of extinction training or the recall of extinction training. To differentiate these two phenomena, it is again important for future work to use as yet unavailable conditional approaches to knock out GRs in astrocytes in the PFC only at the time of extinction training, leaving them intact during extinction recall, and vice versa.

It is important to recognize that our data demonstrate the contributions of GRs in astrocytes of the entire PFC to memory recall. Our injections extended dorsoventrally through both the prelimbic-PFC (PL-PFC) and infralimbic-PFC (IL-PFC). These areas are known to have distinguishable and nuanced contributions to learning, memory and fear expression. First, broadly speaking, the PL-PFC is thought to contribute to the expression of fear and the IL-PFC to the suppression of fear (Giustino and Maren 2015). Second, the PL-PFC has been shown to be important for the learning, consolidation and expression of fear associated with the initial conditioning event, while the IL-PFC is more well known for its role in extinction learning and the inhibition of fear after extinction training (Morgan and LeDoux 1995; Quirk et al. 2000, 2006; Bukalo et al. 2015; Rozeske et al. 2015; Giustino et al. 2019). The knockout of GRs in astrocytes of the PL-PFC could be contributing to the differences in the initial expression of fear during recall of the auditory fear conditioning. In contrast, knockout of GRs in astrocytes of the IL-PFC could be mediating the observed effects on consolidation or recall of extinction training. All of the aforementioned work delineating the role of the PL-PFC and IL-PFC focused on the contributions of neurons. It is possible that astrocytes play a distinct role from the neurons in these regions. Future research will have to determine how GRs in astrocytes specifically of the PL-PFC and IL-PFC contribute to memory recall and compare these contributions with those of GRs in neurons in these regions.

An important dimension of our extinction recall data are the female-specific effects that we report. We do not have any definitive mechanisms via which GRKO in cortical astrocytes impaired extinction recall in female but not male mice. However, we are heartened by a similar pattern that was recently reported. Specifically, knocking out GRs, prior to fear training, in CaMKIIa neurons in the PL-PFC of female but not male rats impaired fear extinction recall and heightened fear expression during training (Scheimann et al. 2019). Females are twice more likely than males to develop PTSD after a traumatic event (Kessler et al. 1995). The female-specific disruption of extinction recall that we report suggest that sex-specific GR expression and action in astrocytes in the IL-PFC might be an attractive molecular candidate to probe further as related to the aforementioned sex-bias in the incidence of PTSD. Sex is already known to influence numbers of astrocytes in brain regions linked to stress responsiveness. For example, male rodents have more astrocytes in the medial amygdala and females have more astrocytes in the hippocampus (Mouton et al. 2002; Johnson et al. 2008; Pfau et al. 2016). Then there are data to suggest that exposure to stress can alter astrocyte biology. In the PFC, chronic stress decreases expression of astrocyte markers (GFAP) and induces atrophy of astrocyte processes in males, while increasing GFAP expression and complexity of astrocyte anatomy in females (Tynan et al. 2013; Bollinger et al. 2019). Such data demonstrate that characteristics of astrocytes at baseline, expression of astrocytic markers, and stress-induced changes in astrocytes are different in males and females. Therefore, it stands to reason that manipulating GRs in astrocytes will differentially change fear expression in the sexes. Motivated by such literature, future experiments will need to compare the relative contributions of GRs in PL-PFC versus IL-PFC astrocytes with a specific focus on sex as a key biological variable that influences astrocyte biology.

Our data measuring extinction recall might leave one with the impression that the AstroGRintact and AstroGRKO males did not demonstrate extinction learning and that the AstroGRKO Females cannot be said to have impaired extinction learning because they show the same high levels of freezing during extinction recall as the male groups. Our findings (Supplemental Table 1) of significant reductions in freezing during the extinction recall test compared with the fear recall test within the individual groups, except the AstroGRKO Females would argue against this interpretation. Combining this supplemental analysis across days (Supplemental Table 1) with the main analysis of data measured on the day of extinction recall (Fig. 3C) highlights that knocking out GRs in astrocytes of the PFC impaired extinction recall in female but not male mice. Finally, the AstroGRintact females display exceptional extinction recall compared with the AstroGRintact males. Albeit not statistically significant (*P* = 0.18), such profiles of sex differences in extinction recall have been observed (Shvil et al. 2014; Clark et al. 2019) and emphasize the utmost importance of pursuing sex differences in learning and memory by including both sexes within the same experimental design.

Recent work has illustrated sex differences in the type of fear responses seen in rodents after fear conditioning. In this study, we primarily analyzed a passive coping strategy, freezing upon presentation of the CS+. However, female rats have been shown to be more likely than males to use an active coping strategy, darting away from perceived danger (Gruene et al. 2015; Colom-Lapetina et al. 2017, 2019). To determine whether active coping could explain our results and complement our freezing measures, darting behavior was scored during the two CS+ presentations of fear memory recall and extinction memory recall (see the Supplemental Material). We did not detect any significant differences in darting behavior between males and females and groups during the CS+ presentations of fear memory recall and extinction memory recall (Supplemental Fig. 4). Of note, we did not find appreciable levels of darting upon presentations of the CS+ like has been observed during and after associative conditioning using rats (Gruene et al. 2015), and consequently it is not surprising that we could not detect differences in active coping strategies that may be dependent on GRs in astrocytes of the PFC. However, the dichotomies of passive versus active coping strategies and sex as well as species differences contained therein are providing new texture to studies of learning and memory, and it will be important for future research to include the study of active avoidance behaviors while manipulating astrocyte specific GRs.

While the relationships between GR signaling and learning, and memory and recall are complicated (Roozendaal 2002; Finsterwald and Alberini 2014), there is evidence to suggest that glucocorticoids (GCs) and GR signaling have a positive influence on these phenomena. For example, glucocorticoid administration has been shown to dampen fear recall and impact consolidation of fear extinction, while blocking GC biosynthesis has been shown to impair extinction of a fear memory (Cai et al. 2006; Blundell et al. 2011). Micro infusions of GCs into the PL-PFC and IL-PFC improved extinction recall and knocking out GRs on neurons of the PL-PFC impaired extinction recall (Reis et al. 2016; Dadkhah et al. 2018; Scheimann et al. 2019). The only study, to our knowledge, that has specifically addressed the role of GRs on astrocytes in extinction showed that knockdown of GRs on astrocytes throughout the brain reduced freezing in a fearful context (Tertil et al. 2018). This whole brain knockdown occurred prior to fear conditioning and, as was interpreted by the authors, suggests that GRs on astrocytes are important for normative memory formation. Similarly, we interpret our work as suggesting that GRs in cortical astrocytes play a role in normative memory recall.

Thinking about how GRs in cortical astrocytes might influence memory recall, we speculate that relationships between GRs and lactate-mediated signaling deserve experimental scrutiny. Not only is astrocyte-neuron lactate signaling important for learning and memory (Suzuki et al. 2011; Gao et al. 2016), but recent work has also demonstrated that GR knockdown in astrocytes decreases neuronal excitability via reduction of GR-induced lactate release (Sada et al. 2015; Skupio et al. 2020). Based on these data, in the future, we propose to test whether the altered memory recall that we report is a consequence of astrocyte GR knockout-induced lactate reduction resulting in reduced neuronal activity in the PFC.

In this paper, we chose to focus specifically on GRs and admittedly ignored the impact of possible compensations in circulating plasma corticosterone levels and mineralocorticoid receptor (MR) action in AstroGRKO animals. Our future work will pay attention to whether circulating corticosterone levels and MR activity are in any way affected by the knockout of GRs in astrocytes of the PFC. Importantly, our data demonstrate that we are still able to detect *nr3c2* (gene encoding MR) transcripts in the PFC of AstroGRKO animals suggesting that our manipulation is specific to GR expression and leaves MR expression intact.

In conclusion, our data provide evidence that GRs on astrocytes are necessary for normative memory recall. To our knowledge, ours is the first report linking GR function specifically in cortical astrocytes to fear-related memory recall. Furthermore, our work highlights GR signaling as a promising avenue of research to explore sex differences in the recall of fear extinction.

## Materials and Methods

### Animals

*Nr3c1*-floxed (GR-floxed) animals were obtained from Jackson Laboratories (strain 012914) and then back-crossed to a C57BL/6J background. homozygous GR-floxed and wild-type littermate controls were group-housed in the vivarium located in the Neuroscience Building of the Yerkes National Primate Research Center and in the Animal Care Facility of the Saban Research Institute, with controlled temperature, humidity and pressure, kept on a 14:10-h light–dark cycle, and given ad libitum access to food and water. Experiments were conducted during the light cycle using adult mice that were ∼3 mo of age. All experiments were approved by the Emory and Children's Hospital Los Angeles Institutional Animal Care and Use Committees and followed NIH standards.

### Auditory fear conditioning

Behavioral sessions were conducted in conditioning chambers (Coulbourn Instruments) connected to tone and shock generators controlled by FreezeFrame software (Actimetrics). On day 1, animals were habituated to the conditioning chambers by placing them in context A for 5 min. Context A consisted of a chamber with metal rod floors, with both the chamber and room lights turned off. Quatricide was used as the cleaning agent. Infrared lights in the chambers were left on to allow for recording. On day 2, animals were conditioned in context A, as follows: After an acclimation period that lasted 180 sec, animals presented with five 30-sec tones (6 kHz, 70 dB), termed the conditioned stimulus (CS+). Each tone presentation coterminated with a 1 sec, 0.5 mA footshock (unconditioned stimulus [US]). Tone-shock pairings were presented with an intertrial interval (ITI) that averaged 2 min. After conditioning, animals were returned to the vivarium for 3–5 d before intracranial surgeries were performed on them as noted below.

### Intracranial surgeries

To knock out GRs in astrocytes of the prefrontal cortex (PFC), GR-floxed mice and littermate controls were injected with an AAV5-GFAP-GFP-Cre virus. These injections resulted in two groups of mice; those in which GRs were left intact in astrocytes in the PFC (AstroGRintact) and those in which GRs were knocked out in astrocytes in the PFC (AstroGRKO). Viruses were purchased from Addgene. Animals were given oral Metacam (meloxicam) as an analgesic and then anesthetized using a mixture of ketamine and dexdomitor, injected interperitoneally. Animals were mounted into stereotaxic frames (Stoelting) and stereotaxic injections were performed bilaterally into the PFC at the following coordinates relative to Bregma: AP +1.69 mm, ML ±0.16 mm, DV −2.85 mm. Viruses were injected using a Nanoject III (Drummond Scientific) with a pulled glass micropipette. The final volume of AAV-containing solution was 80 nL, injected at a rate of 1 nL/sec. Following injections, the glass pipettes were left in place for 5 min before slowly being removed over 1 min. The scalp was closed with sutures and the animal was administered Antisedan, to counteract the anesthesia. Animals were moved back to the vivarium to recover. Further behavioral testing was conducted 2 wk later to allow for optimal expression of the CRE recombinase and/or fluorescent protein.

### Testing recall of fear memory and memory of extinction training

After viral expression (2 wk postsurgery), animals’ recall of fear memory and of extinction training was measured as follows. Briefly, after 180 sec of acclimation to the same chambers as conditioning, but with distinct contextual indicators (context B), animals were exposed to 30 CS+ tone presentations with 30 sec ITI between CS+ presentations. Specifically, the chambers were fitted with opaque plexiglass floors, both the house lights and room lights were on, and 70% ethanol was used to clean the chambers. Recall of fear memory was measured by averaging freezing during the first 2 CS+ tone presentations. Within-session extinction was measured as the freezing responses to the CS+ presentations in bins of five across the entire 30 presentations of the CS+ tones. One day later, recall of extinction memory was measured by presenting 2 CS+ tones in context B without any associated footshock.

### Behavioral analyses

All behavior was video recorded and the total amount of time spent freezing (in seconds) to the tones was analyzed using FreezeFrame software (Actimetrics) by an experimenter blind to the treatment conditions. Freezing was defined as no change in velocity for >0.5 sec.

### Histology

To confirm the expression and placement of intracranial virus injections, animals were anesthetized and *trans*-cardially perfused using a 4% paraformaldehyde/phosphate-buffered saline solution (PFA/PBS). Brains were stored overnight in 4% PFA/PBS and then transferred to a 30% sucrose/PBS solution until saturated (3–4 d). Brains were then sectioned at 35 µm on a freezing microtome (Leica), stained with Hoechst nuclear stain (1:1000), and mounted with SlowFade Gold Antifade mounting solution (Life Technologies). Location of the GFP fluorescent tag was observed with a Nikon Eclipse E800 fluorescent microscope and used to determine virus placement and expression.

### Fluorescence-activated cell sorting (FACS) and RT-qPCR to determine GR knockout in astrocytes

#### Animal brain dissociation

AstroGRintact and AstroGRKO mice were sacrificed after isoflurane anesthesia and whole brains were isolated. Brains was blocked to isolate an ∼300-µm brain section that contained the prefrontal cortex (PFC). The PFC was punched using a 1mm stainless steel tissue punch. The Papain dissociation kit (Worthington LK003150) was used to dissociate the tissue. Briefly, tissue was minced 100 times on glass slide and then added to 1 mL of Worthington papain solution. The tissue was then placed in a shaking incubator for 30 min at 37°C and triturated every 5 min with a fire-polished 5-mL glass Pasteur pipette. Undissociated tissue was allowed to settle, and supernatant was placed in a new tube and centrifuged at 300*g* for 5 min at 4°C. The cell pellet was suspended in 1 mL of Worthington albumin-ovomucoid inhibitor solution for 5 min at 4°C. The resulting solution was centrifuged at 300*g* for 5 min at 4°C and the pellet was suspended in 0.5 mL of PBS 1% FBS FACS solution.

#### FACS sorting

Dissociated brain cells were filtered into FACS tubes with filter tops (BD 352235) to eliminate clumps and then FACS sorted using a FACS ARIA II FACS sorter. Cells were gated using forward scatter (FSC-A) and side scatter (SSC-A). Clumps and doublets were removed by gating singlets in two linear scale dot-plots of SSC-W versus SSC-H and FSC-W and FSC-H. GFP positive cells were sorted based on fluorescence intensity and size of cells and gated. Approximately 20,000 cells were collected at 4°C in 1.5-mL microcentrifuge tubes containing PBS 1% FBS FACS solution. Cells were pelleted for RNA Extraction.

#### RNA extraction and qRT-PCR

RNA for the FACS sorted cells was extracted using the Pico pure RNA isolation kit (Arcturus kit0204). Purification was performed according to the manufacturer's protocol using RNA extraction from cell pellet protocol with on-column DNase treatment and eluted with 12 µL of elution buffer. cDNA was synthesized from 40 ng of isolated RNA using the RT^2^ first strand kit (Qiagen 330404). Preamplification of cDNA was done using single cell to CT kit (Thermo Fisher Scientific 445 8237) and TaqMan gene expression assay primer sets (*gapdh* [Mm99999915_g1], *nr3c1* [Mm00433832_m1], and *nr3c2* [Mm01241596_m1]). Standard qRT-PCR was done using TaqMan 2× universal mix (ABI 430 4437) with same primer sets on an Applied Biosystem 7500 Fast real-time PCR system.

### Statistics

Statistics were performed with GraphPad Prism. Repeated-measures two-way ANOVA (GR status and CS) was used for behavioral analysis of fear conditioning and extinction training. Two-way ANOVA (GR status and sex) was used to analyze the memory recall data that is, recall of fear memory and recall of extinction. Sidak comparisons were used for post-hoc analyses. Significance for all experiments was set at *P* < 0.05.

## Supplementary Material

Supplemental Material
