# Supplementary material for: Contributions of glucocorticoid receptors in cortical astrocytes to memory recall
Source: Learn Mem. 2021 Apr;28(4):126–33. doi: 10.1101/lm.053041.120 (PMC7970741; doi:10.1101/lm.053041.120)
Supplement: Supplemental Material [file supp_28_4_126__index.html]

Supplemental Material 

# Contributions of glucocorticoid receptors in cortical astrocytes to memory recall

## Supplemental Material

- Supplemental\_Fig\_1.docx
- Supplemental\_Fig\_2.docx
- Supplemental\_Fig\_3.docx
- Supplemental\_Fig\_4.docx
- Supplemental\_Methods.docx
- Supplemental\_Table\_1.docx
