# Supplementary material for: Contributions of glucocorticoid receptors in cortical astrocytes to memory recall
Source: Learn Mem. 2021 Apr;28(4):126–33. doi: 10.1101/lm.053041.120 (PMC7970741; doi:10.1101/lm.053041.120)
Supplement: Supplemental Material [file supp_28.4.126_Supplemental_Fig_1.docx]

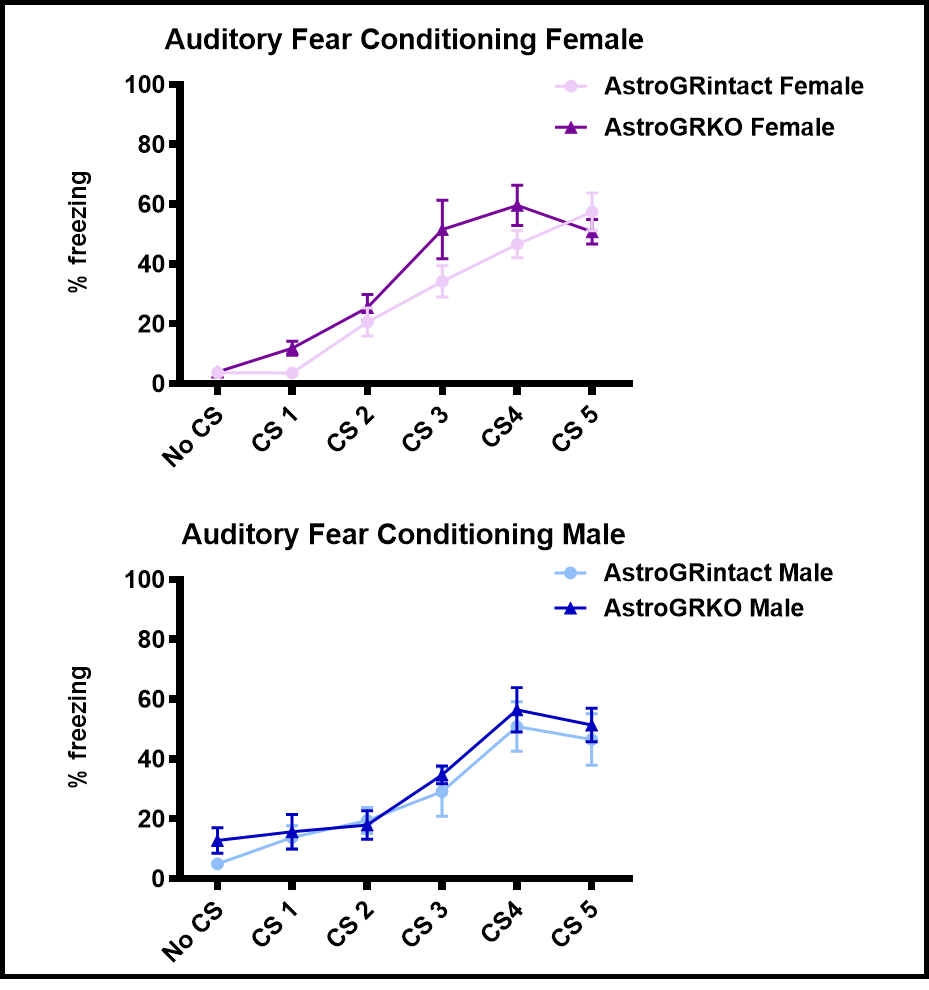


**Supplementary Fig 1.**

**Prior to loss of GRs in cortical astrocytes all groups acquire fear to the CS+ similarly.** Prior to GRs being knocked-out in the PFC of AstroGRKO mice (which occurs after fear conditioning), AstroGRKO mice showed no differences in the acquisition of fear during auditory fear conditioning. Data shown are split by sex, show percent time freezing to the CS+, and are represented as Mean±SEM.
