# Supplementary material for: Contributions of glucocorticoid receptors in cortical astrocytes to memory recall
Source: Learn Mem. 2021 Apr;28(4):126–33. doi: 10.1101/lm.053041.120 (PMC7970741; doi:10.1101/lm.053041.120)
Supplement: Supplemental Material [file supp_28.4.126_Supplemental_Fig_2.docx]

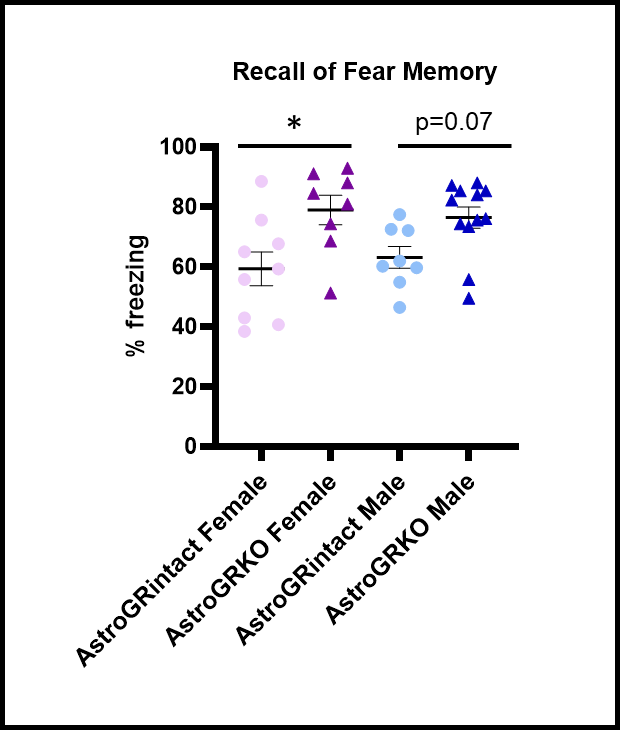


**Supplementary Fig 2.**

**Loss of GRs in cortical astrocytes disrupts normative fear recall in both male and female mice.** Following auditory fear conditioning and knock-out of GRs in astrocytes in the PFC, freezing to the first two presentations of the CS+ was used to assess recall of fear memory. AstroGRKO animals froze more (Fig. 2C), with no significant interaction between sex and GR status. Post-hoc multiple comparisons test showed a significant difference between AstroGRintact and AstroGRKO females (p=0.01) and a similar trend in males (AstroGRintact vs AstroGRKO – Male p = 0.07). Data are split by sex, show average percent time freezing to the first two CS+ presentations, and are represented as Mean+SEM.
