# Supplementary material for: Contributions of glucocorticoid receptors in cortical astrocytes to memory recall
Source: Learn Mem. 2021 Apr;28(4):126–33. doi: 10.1101/lm.053041.120 (PMC7970741; doi:10.1101/lm.053041.120)
Supplement: Supplemental Material [file supp_28.4.126_Supplemental_Fig_3.docx]

**
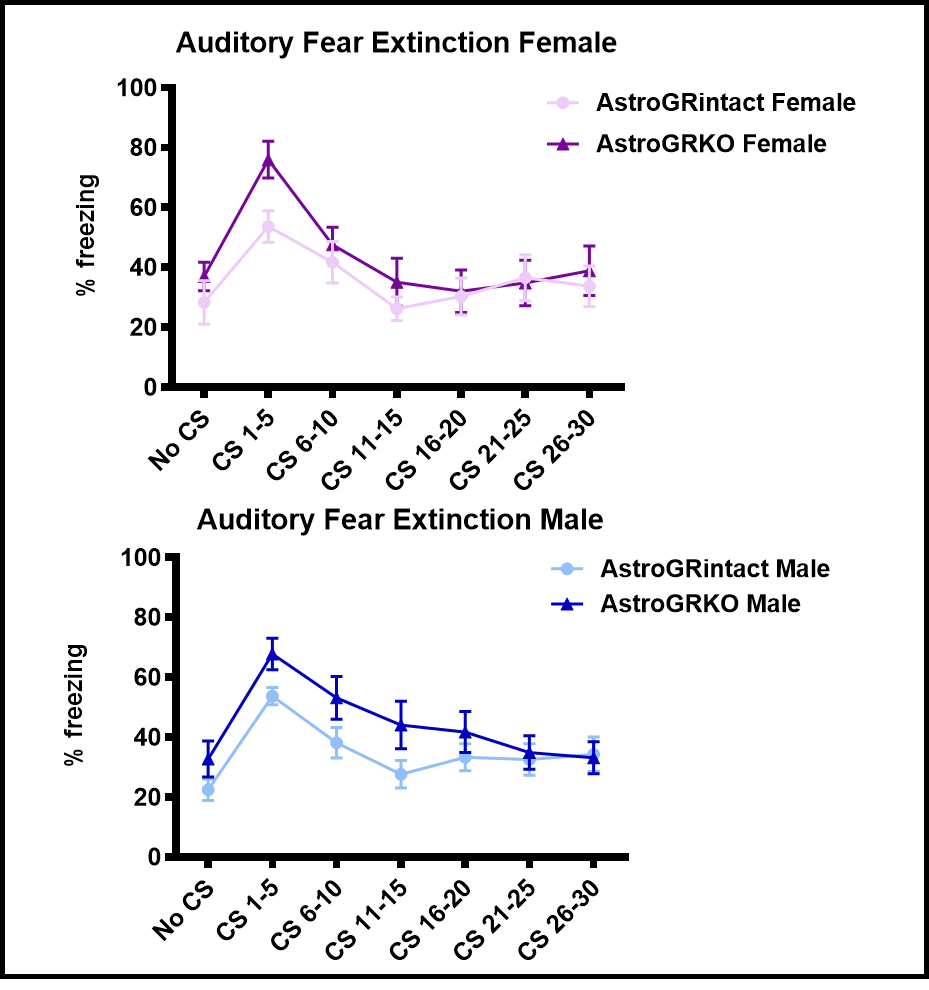
**

**Supplementary Fig 3.**

**Loss of GRs in cortical astrocytes does not affect acquisition of extinction.** Following GRs being knocked-out in astrocytes in the PFC after auditory fear conditioning, AstroGRKO mice show no differences in freezing to the CS+ during repeated non-reinforced presentations of the CS+ during extinction training when compared to AstroGRintact mice. Data are split by sex and show percent time freezing to 30 CS+ presentations (6 bins of 5 CS+ presentations) and are represented as Mean±SEM.
