# Supplementary material for: Contributions of glucocorticoid receptors in cortical astrocytes to memory recall
Source: Learn Mem. 2021 Apr;28(4):126–33. doi: 10.1101/lm.053041.120 (PMC7970741; doi:10.1101/lm.053041.120)
Supplement: Supplemental Material [file supp_28.4.126_Supplemental_Fig_4.docx]

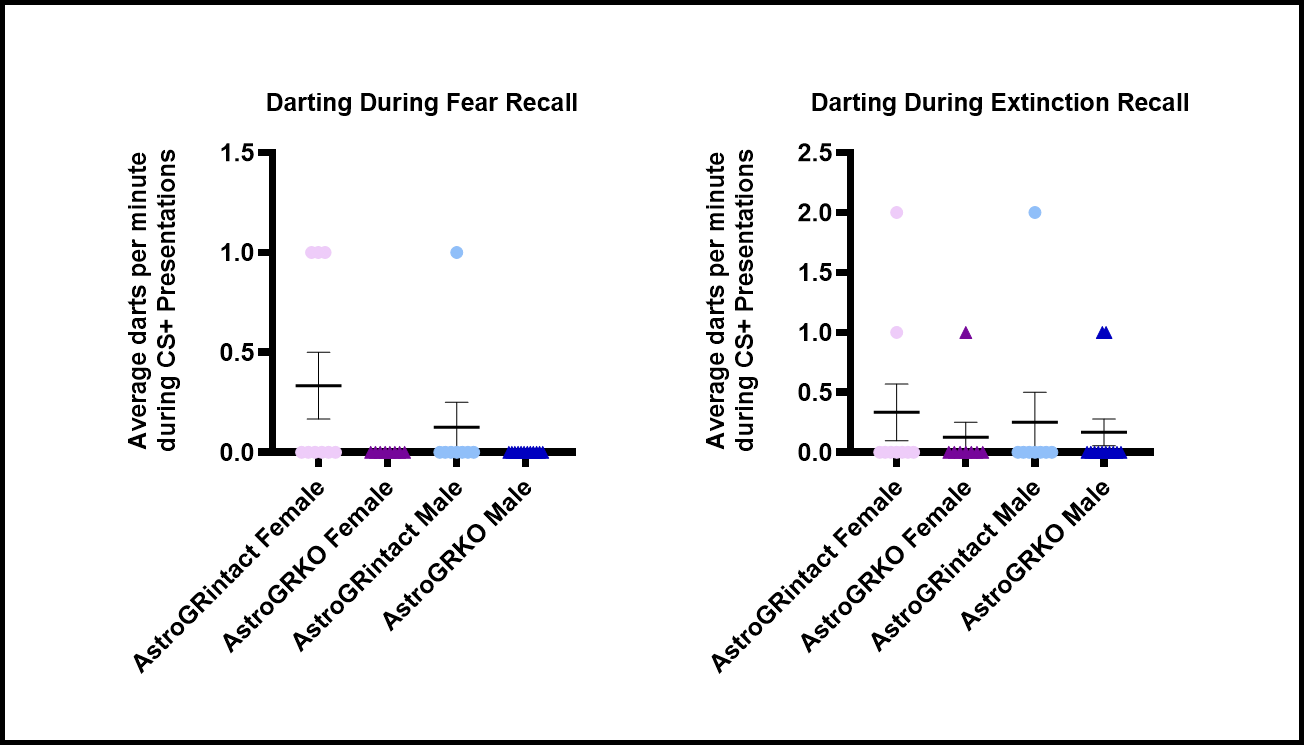


**Supplementary Fig 4.**

**Loss of GRs in cortical astrocytes does not affect darting behavior.** While testing for fear memory recall, we did not find any differences in darting behavior between groups during the CS+ presentations (Kruskall-Wallis Test: H(3) = 6.989, p > 0.05). Similarly, while testing for recall of extinction training, we did not find any differences in darting behavior between groups during the CS+ presentations (Kruskall-Wallis Test: H(3) = 0.423, p > 0.05).
