# Supplementary material for: Contributions of glucocorticoid receptors in cortical astrocytes to memory recall
Source: Learn Mem. 2021 Apr;28(4):126–33. doi: 10.1101/lm.053041.120 (PMC7970741; doi:10.1101/lm.053041.120)
Supplement: Supplemental Material [file supp_28.4.126_Supplemental_Methods.docx]

**SUPPLEMENTARY METHODS.**

**Darting active avoidance behavior:** Videos taken during fear recall and extinction recall testing were hand-scored for darting behavior by an investigator blind to the treatment conditions. Darting was defined as a rapid movement across the chamber during a period that the animal was attending to the CS+ (freezing). Any rapid movements as a part of exploratory behavior were ignored.
