# Supplementary material for: Contributions of glucocorticoid receptors in cortical astrocytes to memory recall
Source: Learn Mem. 2021 Apr;28(4):126–33. doi: 10.1101/lm.053041.120 (PMC7970741; doi:10.1101/lm.053041.120)
Supplement: Supplemental Material [file supp_28.4.126_Supplemental_Table_1.docx]

| Average Freezing ± SEM and T-test Comparison | | |
| --- | --- | --- |
|  | Fear Recall | Extinction Recall |
| AstroGRintact Female | 59.34 ± 5.63 | 38.94 ± 7.52 |
| p value | 0.008 | |
| AstroGRKO Female | 78.98 ± 4.92 | 63.20 ± 7.52 |
| p value | 0.1 | |
| AstroGRintact Male | 63.16 ± 3.64 | 51.99 ± 4.55 |
| p value | 0.03 | |
| AstroGRKO Male | 76.44 ± 3.55 | 54.57 ± 3.90 |
| p value | 0.0004 | |

**Supplementary Table 1:** **Comparison of freezing to CS+ while testing recall of fear memory (after conditioning) and recall of extinction training (after extinction).** AstroGRintact Female, AstroGRintact Male and AstroGRKO Male, but not AstroGRKO Female mice show evidence of extinction learning when comparing their freezing to the CS+ during the test for recall of fear memory after conditioning (Fear Recall) with their freezing to the CS+ during the test for recall of extinction training (Extinction Recall). Data represented as Mean±SEM.
